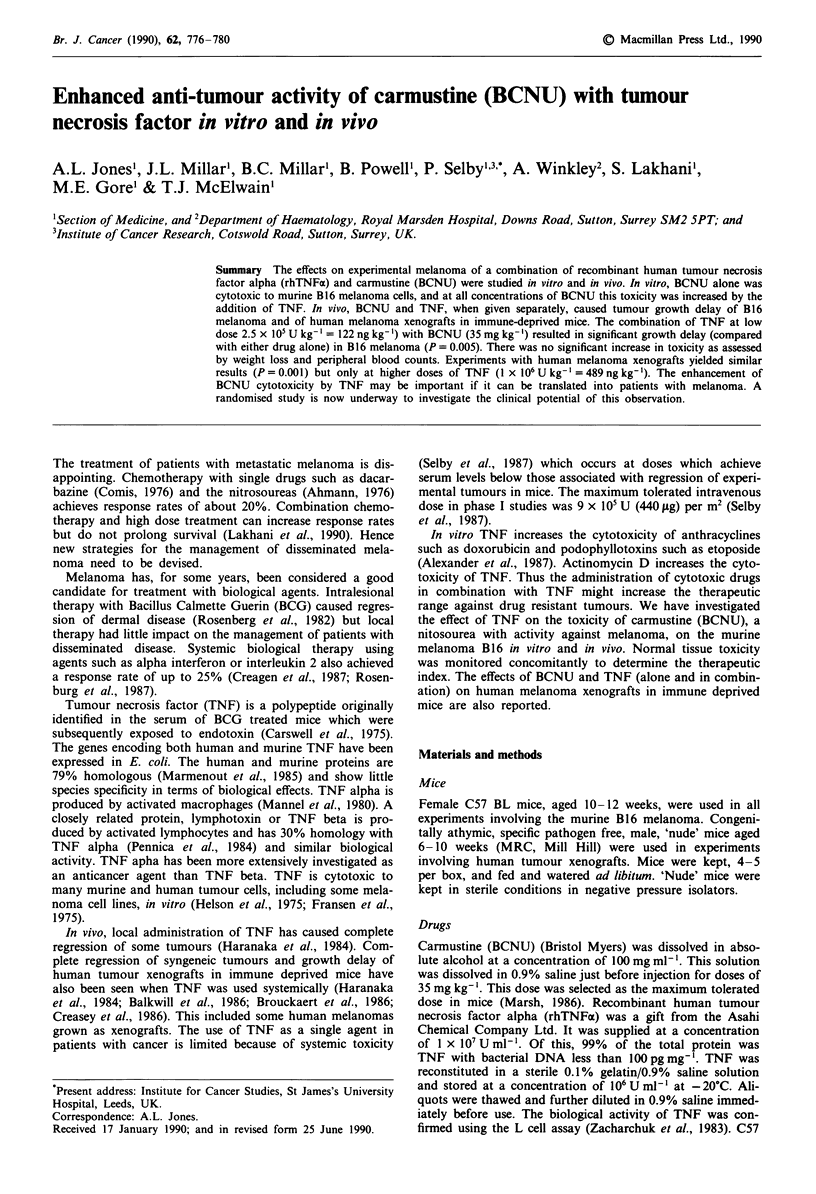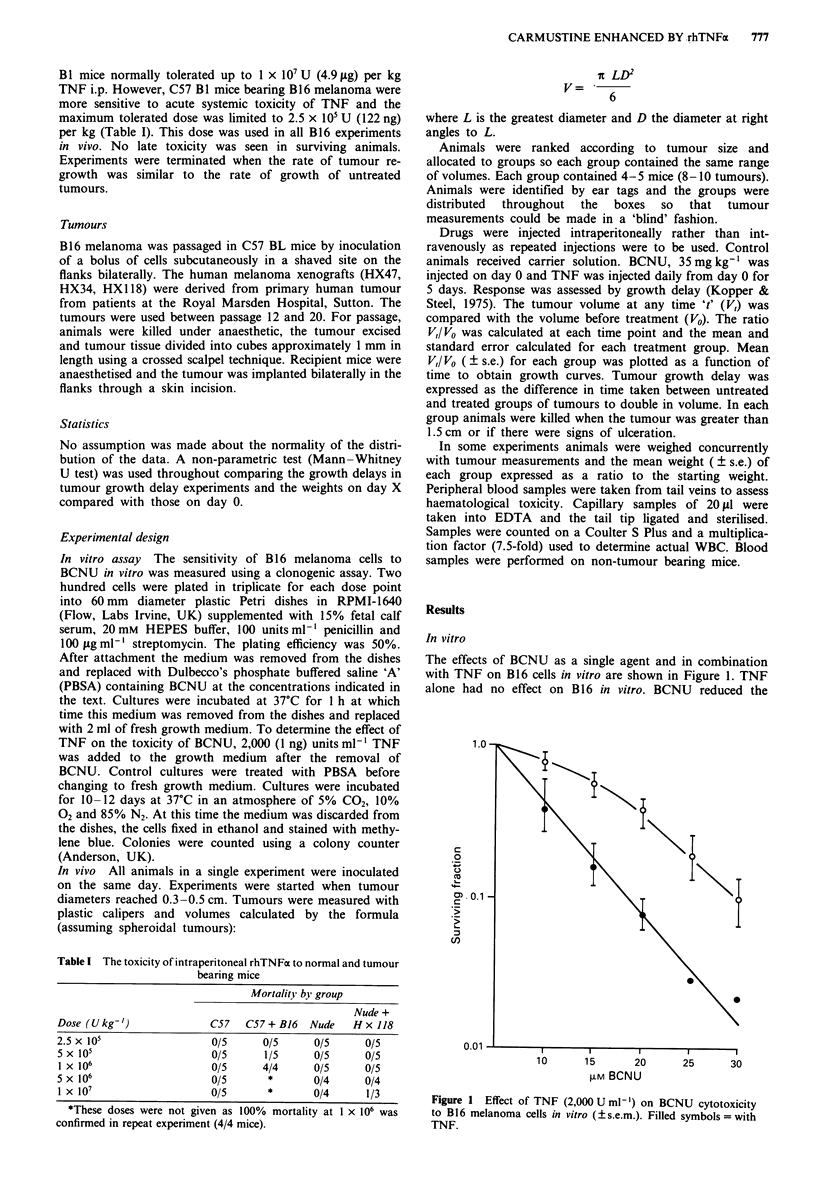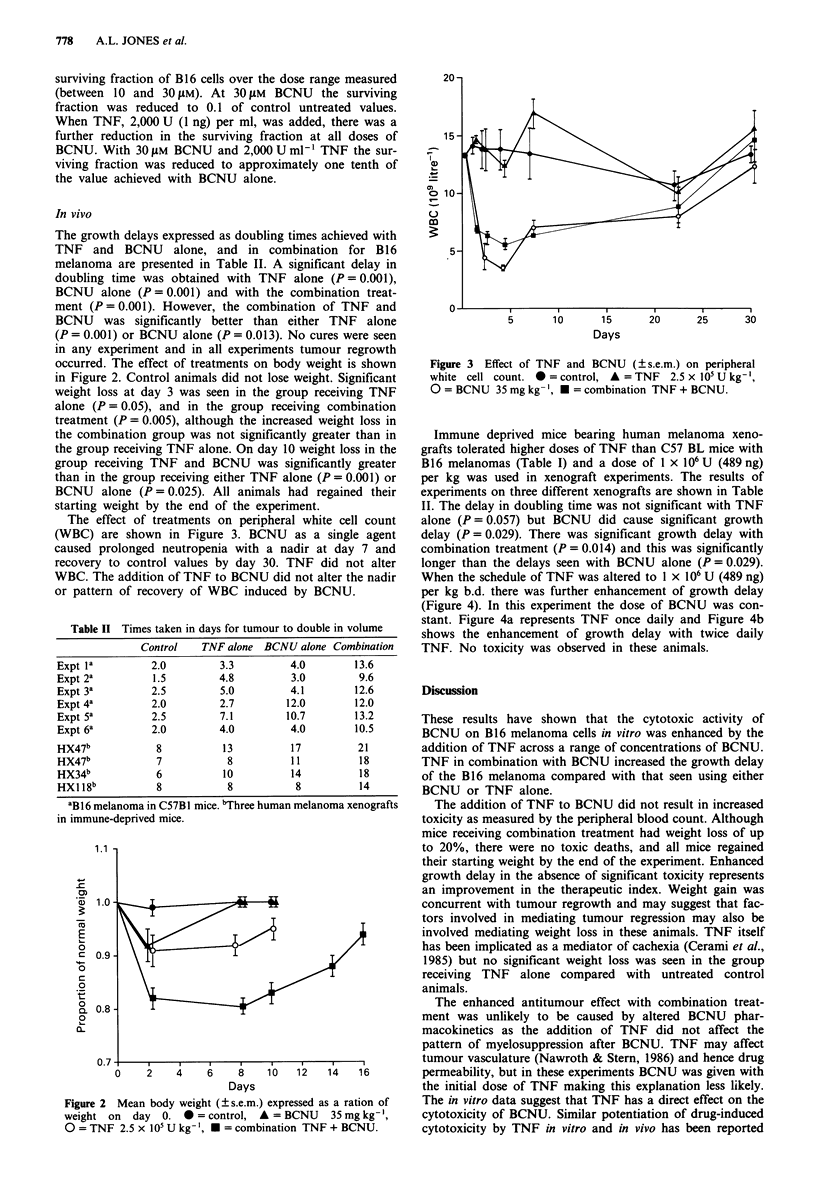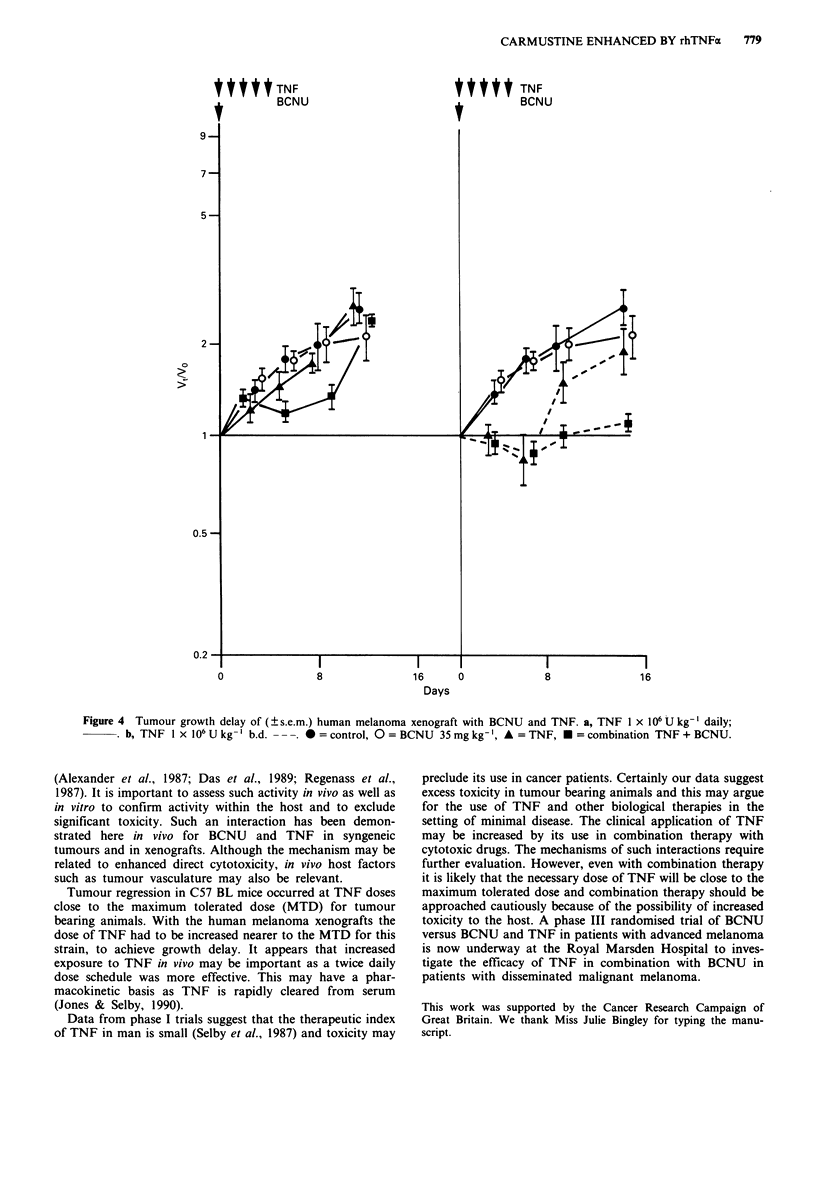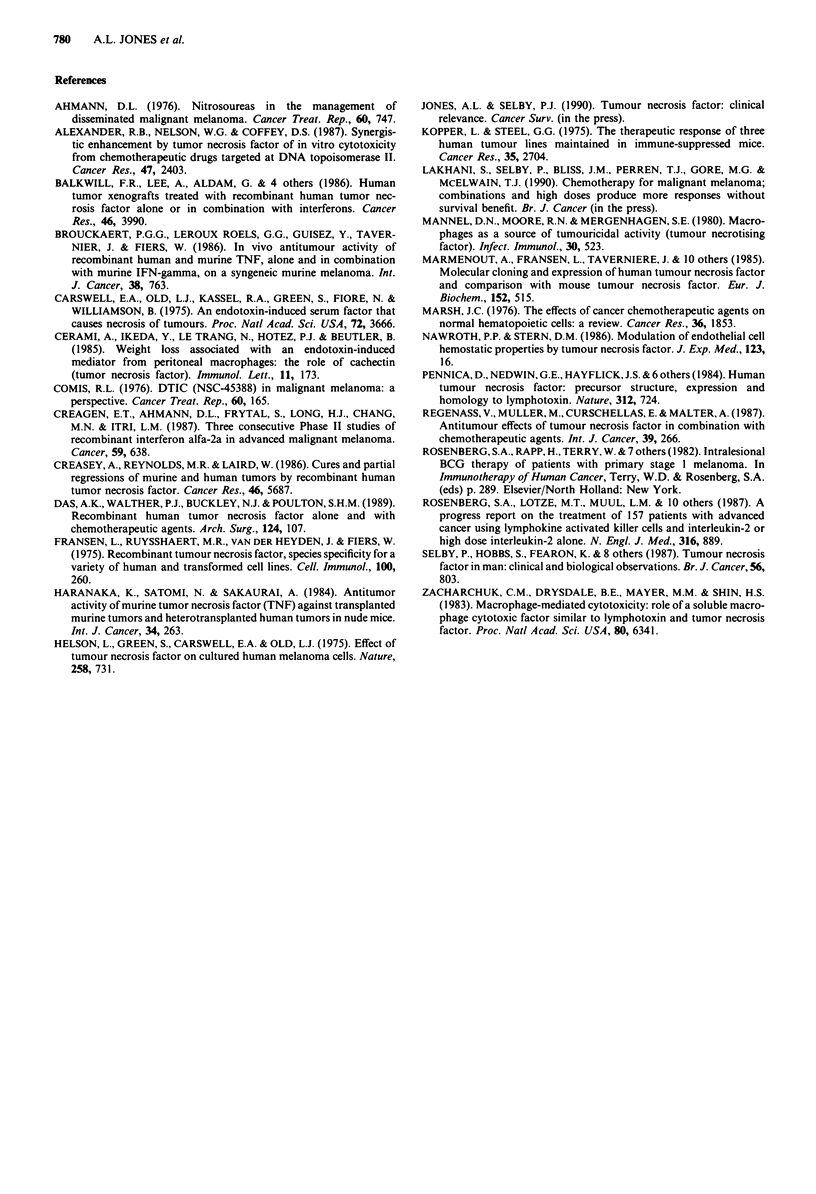# Enhanced anti-tumour activity of carmustine (BCNU) with tumour necrosis factor in vitro and in vivo.

**DOI:** 10.1038/bjc.1990.378

**Published:** 1990-11

**Authors:** A. L. Jones, J. L. Millar, B. C. Millar, B. Powell, P. Selby, A. Winkley, S. Lakhani, M. E. Gore, T. J. McElwain

**Affiliations:** Section of Medicine, Royal Marsden Hospital, Sutton, Surrey, UK.

## Abstract

The effects on experimental melanoma of a combination of recombinant human tumour necrosis factor alpha (rhTNF alpha) and carmustine (BCNU) were studied in vitro and in vivo. In vitro, BCNU alone was cytotoxic to murine B16 melanoma cells, and at all concentrations of BCNU this toxicity was increased by the addition of TNF. In vivo, BCNU and TNF, when given separately, caused tumour growth delay of B16 melanoma and of human melanoma xenografts in immune-deprived mice. The combination of TNF at low dose 2.5 x 10(5) U kg-1 = 122 ng kg-1) with BCNU (35 mg kg-1) resulted in significant growth delay (compared with either drug alone) in B16 melanoma (P = 0.005). There was no significant increase in toxicity as assessed by weight loss and peripheral blood counts. Experiments with human melanoma xenografts yielded similar results (P = 0.001) but only at higher doses of TNF (1 x 10(6) U kg-1 = 489 ng kg-1). The enhancement of BCNU cytotoxicity by TNF may be important if it can be translated into patients with melanoma. A randomised study is now underway to investigate the clinical potential of this observation.


Br. J. Cancer (1990), 62, 776-780 © Macmillan Press Ltd., 1990

### Enhanced anti-tumour activity of carmustine (BCNU) with tumour necrosis factor *in vitro* and *in vivo*

A.L. Jones[1], J.L. Millar[1], B.C. Millar[1], B. Powell[1], P. Selby[1,3,*], A. Winkley[2], S. Lakhani[1], M.E. Gore[1] & T.J. McElwain[1]

[1]*Section of Medicine, and* [2]*Department of Haematology, Royal Marsden Hospital, Downs Road, Sutton, Surrey SM2 5PT; and* [3]*Institute of Cancer Research, Cotswold Road, Sutton, Surrey, UK.*

**Summary** The effects on experimental melanoma of a combination of recombinant human tumour necrosis factor alpha (rhTNFα) and carmustine (BCNU) were studied *in vitro* and *in vivo*. *In vitro*, BCNU alone was cytotoxic to murine B16 melanoma cells, and at all concentrations of BCNU this toxicity was increased by the addition of TNF. *In vivo*, BCNU and TNF, when given separately, caused tumour growth delay of B16 melanoma and of human melanoma xenografts in immune-deprived mice. The combination of TNF at low dose $2.5 \times 10^5$ U kg$^{-1}$ = 122 ng kg$^{-1}$) with BCNU (35 mg kg$^{-1}$) resulted in significant growth delay (compared with either drug alone) in B16 melanoma ($P = 0.005$). There was no significant increase in toxicity as assessed by weight loss and peripheral blood counts. Experiments with human melanoma xenografts yielded similar results ($P = 0.001$) but only at higher doses of TNF ($1 \times 10^6$ U kg$^{-1}$ = 489 ng kg$^{-1}$). The enhancement of BCNU cytotoxicity by TNF may be important if it can be translated into patients with melanoma. A randomised study is now underway to investigate the clinical potential of this observation.

The treatment of patients with metastatic melanoma is disappointing. Chemotherapy with single drugs such as dacarbazine (Comis, 1976) and the nitrosoureas (Ahmann, 1976) achieves response rates of about 20%. Combination chemotherapy and high dose treatment can increase response rates but do not prolong survival (Lakhani *et al.*, 1990). Hence new strategies for the management of disseminated melanoma need to be devised.

Melanoma has, for some years, been considered a good candidate for treatment with biological agents. Intralesional therapy with Bacillus Calmette Guerin (BCG) caused regression of dermal disease (Rosenberg *et al.*, 1982) but local therapy had little impact on the management of patients with disseminated disease. Systemic biological therapy using agents such as alpha interferon or interleukin 2 also achieved a response rate of up to 25% (Creagen *et al.*, 1987; Rosenburg *et al.*, 1987).

Tumour necrosis factor (TNF) is a polypeptide originally identified in the serum of BCG treated mice which were subsequently exposed to endotoxin (Carswell *et al.*, 1975). The genes encoding both human and murine TNF have been expressed in *E. coli*. The human and murine proteins are 79% homologous (Marmenout *et al.*, 1985) and show little species specificity in terms of biological effects. TNF alpha is produced by activated macrophages (Mannel *et al.*, 1980). A closely related protein, lymphotoxin or TNF beta is produced by activated lymphocytes and has 30% homology with TNF alpha (Pennica *et al.*, 1984) and similar biological activity. TNF apha has been more extensively investigated as an anticancer agent than TNF beta. TNF is cytotoxic to many murine and human tumour cells, including some melanoma cell lines, *in vitro* (Helson *et al.*, 1975; Fransen *et al.*, 1975).

*In vivo*, local administration of TNF has caused complete regression of some tumours (Haranaka *et al.*, 1984). Complete regression of syngeneic tumours and growth delay of human tumour xenografts in immune deprived mice have also been seen when TNF was used systemically (Haranaka *et al.*, 1984; Balkwill *et al.*, 1986; Brouckaert *et al.*, 1986; Creasey *et al.*, 1986). This included some human melanomas grown as xenografts. The use of TNF as a single agent in patients with cancer is limited because of systemic toxicity (Selby *et al.*, 1987) which occurs at doses which achieve serum levels below those associated with regression of experimental tumours in mice. The maximum tolerated intravenous dose in phase I studies was $9 \times 10^5$ U (440 μg) per m$^2$ (Selby *et al.*, 1987).

*In vitro* TNF increases the cytotoxicity of anthracyclines such as doxorubicin and podophyllotoxins such as etoposide (Alexander *et al.*, 1987). Actinomycin D increases the cytotoxicity of TNF. Thus the administration of cytotoxic drugs in combination with TNF might increase the therapeutic range against drug resistant tumours. We have investigated the effect of TNF on the toxicity of carmustine (BCNU), a nitosourea with activity against melanoma, on the murine melanoma B16 *in vitro* and *in vivo*. Normal tissue toxicity was monitored concomitantly to determine the therapeutic index. The effects of BCNU and TNF (alone and in combination) on human melanoma xenografts in immune deprived mice are also reported.

### Materials and methods

### *Mice*

Female C57 BL mice, aged 10-12 weeks, were used in all experiments involving the murine B16 melanoma. Congenitally athymic, specific pathogen free, male, 'nude' mice aged 6-10 weeks (MRC, Mill Hill) were used in experiments involving human tumour xenografts. Mice were kept, 4-5 per box, and fed and watered *ad libitum*. 'Nude' mice were kept in sterile conditions in negative pressure isolators.

### *Drugs*

Carmustine (BCNU) (Bristol Myers) was dissolved in absolute alcohol at a concentration of 100 mg ml$^{-1}$. This solution was dissolved in 0.9% saline just before injection for doses of 35 mg kg$^{-1}$. This dose was selected as the maximum tolerated dose in mice (Marsh, 1986). Recombinant human tumour necrosis factor alpha (rhTNFα) was a gift from the Asahi Chemical Company Ltd. It was supplied at a concentration of $1 \times 10^7$ U ml$^{-1}$. Of this, 99% of the total protein was TNF with bacterial DNA less than 100 pg mg$^{-1}$. TNF was reconstituted in a sterile 0.1% gelatin/0.9% saline solution and stored at a concentration of $10^6$ U ml$^{-1}$ at −20°C. Aliquots were thawed and further diluted in 0.9% saline immediately before use. The biological activity of TNF was confirmed using the L cell assay (Zacharchuk *et al.*, 1983). C57

*Present address: Institute for Cancer Studies, St James's University Hospital, Leeds, UK.
Correspondence: A.L. Jones.
Received 17 January 1990; and in revised form 25 June 1990.

Bl mice normally tolerated up to $1 \times 10^7$ U (4.9 µg) per kg TNF i.p. However, C57 Bl mice bearing B16 melanoma were more sensitive to acute systemic toxicity of TNF and the maximum tolerated dose was limited to $2.5 \times 10^5$ U (122 ng) per kg (Table I). This dose was used in all B16 experiments *in vivo*. No late toxicity was seen in surviving animals. Experiments were terminated when the rate of tumour regrowth was similar to the rate of growth of untreated tumours.

### Tumours

B16 melanoma was passaged in C57 BL mice by inoculation of a bolus of cells subcutaneously in a shaved site on the flanks bilaterally. The human melanoma xenografts (HX47, HX34, HX118) were derived from primary human tumour from patients at the Royal Marsden Hospital, Sutton. The tumours were used between passage 12 and 20. For passage, animals were killed under anaesthetic, the tumour excised and tumour tissue divided into cubes approximately 1 mm in length using a crossed scalpel technique. Recipient mice were anaesthetised and the tumour was implanted bilaterally in the flanks through a skin incision.

### Statistics

No assumption was made about the normality of the distribution of the data. A non-parametric test (Mann-Whitney U test) was used throughout comparing the growth delays in tumour growth delay experiments and the weights on day X compared with those on day 0.

### Experimental design

*In vitro assay* The sensitivity of B16 melanoma cells to BCNU *in vitro* was measured using a clonogenic assay. Two hundred cells were plated in triplicate for each dose point into 60 mm diameter plastic Petri dishes in RPMI-1640 (Flow, Labs Irvine, UK) supplemented with 15% fetal calf serum, 20 mM HEPES buffer, 100 units $ml^{-1}$ penicillin and 100 µg $ml^{-1}$ streptomycin. The plating efficiency was 50%. After attachment the medium was removed from the dishes and replaced with Dulbecco's phosphate buffered saline 'A' (PBSA) containing BCNU at the concentrations indicated in the text. Cultures were incubated at 37°C for 1 h at which time this medium was removed from the dishes and replaced with 2 ml of fresh growth medium. To determine the effect of TNF on the toxicity of BCNU, 2,000 (1 ng) units $ml^{-1}$ TNF was added to the growth medium after the removal of BCNU. Control cultures were treated with PBSA before changing to fresh growth medium. Cultures were incubated for 10-12 days at 37°C in an atmosphere of 5% $CO_2$, 10% $O_2$ and 85% $N_2$. At this time the medium was discarded from the dishes, the cells fixed in ethanol and stained with methylene blue. Colonies were counted using a colony counter (Anderson, UK).

*In vivo* All animals in a single experiment were inoculated on the same day. Experiments were started when tumour diameters reached 0.3-0.5 cm. Tumours were measured with plastic calipers and volumes calculated by the formula (assuming spheroidal tumours):

$$V = \frac{\pi LD^2}{6}$$

where $L$ is the greatest diameter and $D$ the diameter at right angles to $L$.

Animals were ranked according to tumour size and allocated to groups so each group contained the same range of volumes. Each group contained 4-5 mice (8-10 tumours). Animals were identified by ear tags and the groups were distributed throughout the boxes so that tumour measurements could be made in a 'blind' fashion.

Drugs were injected intraperitoneally rather than intravenously as repeated injections were to be used. Control animals received carrier solution. BCNU, 35 mg $kg^{-1}$ was injected on day 0 and TNF was injected daily from day 0 for 5 days. Response was assessed by growth delay (Kopper & Steel, 1975). The tumour volume at any time '$t$' ($V_t$) was compared with the volume before treatment ($V_0$). The ratio $V_t/V_0$ was calculated at each time point and the mean and standard error calculated for each treatment group. Mean $V_t/V_0$ ($\pm$ s.e.) for each group was plotted as a function of time to obtain growth curves. Tumour growth delay was expressed as the difference in time taken between untreated and treated groups of tumours to double in volume. In each group animals were killed when the tumour was greater than 1.5 cm or if there were signs of ulceration.

In some experiments animals were weighed concurrently with tumour measurements and the mean weight ($\pm$ s.e.) of each group expressed as a ratio to the starting weight. Peripheral blood samples were taken from tail veins to assess haematological toxicity. Capillary samples of 20 µl were taken into EDTA and the tail tip ligated and sterilised. Samples were counted on a Coulter S Plus and a multiplication factor (7.5-fold) used to determine actual WBC. Blood samples were performed on non-tumour bearing mice.

**Table I** The toxicity of intraperitoneal rhTNFα to normal and tumour bearing mice

| | *Mortality by group* | | | |
|---|---|---|---|---|
| *Dose (U $kg^{-1}$)* | *C57* | *C57 + B16* | *Nude* | *Nude + H × 118* |
| $2.5 \times 10^5$ | 0/5 | 0/5 | 0/5 | 0/5 |
| $5 \times 10^5$ | 0/5 | 1/5 | 0/5 | 0/5 |
| $1 \times 10^6$ | 0/5 | 4/4 | 0/5 | 0/5 |
| $5 \times 10^6$ | 0/5 | * | 0/4 | 0/4 |
| $1 \times 10^7$ | 0/5 | * | 0/4 | 1/3 |

*These doses were not given as 100% mortality at $1 \times 10^6$ was confirmed in repeat experiment (4/4 mice).

### Results

### In vitro

The effects of BCNU as a single agent and in combination with TNF on B16 cells *in vitro* are shown in Figure 1. TNF alone had no effect on B16 *in vitro*. BCNU reduced the

**Figure 1** Effect of TNF (2,000 U $ml^{-1}$) on BCNU cytotoxicity to B16 melanoma cells *in vitro* ($\pm$s.e.m.). Filled symbols = with TNF.

surviving fraction of B16 cells over the dose range measured (between 10 and 30 μM). At 30 μM BCNU the surviving fraction was reduced to 0.1 of control untreated values. When TNF, 2,000 U (1 ng) per ml, was added, there was a further reduction in the surviving fraction at all doses of BCNU. With 30 μM BCNU and 2,000 U $ml^{-1}$ TNF the surviving fraction was reduced to approximately one tenth of the value achieved with BCNU alone.

### *In vivo*

The growth delays expressed as doubling times achieved with TNF and BCNU alone, and in combination for B16 melanoma are presented in Table II. A significant delay in doubling time was obtained with TNF alone ($P = 0.001$), BCNU alone ($P = 0.001$) and with the combination treatment ($P = 0.001$). However, the combination of TNF and BCNU was significantly better than either TNF alone ($P = 0.001$) or BCNU alone ($P = 0.013$). No cures were seen in any experiment and in all experiments tumour regrowth occurred. The effect of treatments on body weight is shown in Figure 2. Control animals did not lose weight. Significant weight loss at day 3 was seen in the group receiving TNF alone ($P = 0.05$), and in the group receiving combination treatment ($P = 0.005$), although the increased weight loss in the combination group was not significantly greater than in the group receiving TNF alone. On day 10 weight loss in the group receiving TNF and BCNU was significantly greater than in the group receiving either TNF alone ($P = 0.001$) or BCNU alone ($P = 0.025$). All animals had regained their starting weight by the end of the experiment.

The effect of treatments on peripheral white cell count (WBC) are shown in Figure 3. BCNU as a single agent caused prolonged neutropenia with a nadir at day 7 and recovery to control values by day 30. TNF did not alter WBC. The addition of TNF to BCNU did not alter the nadir or pattern of recovery of WBC induced by BCNU.

**Table II** Times taken in days for tumour to double in volume

| | *Control* | *TNF alone* | *BCNU alone* | *Combination* |
|---|---|---|---|---|
| Expt 1[a] | 2.0 | 3.3 | 4.0 | 13.6 |
| Expt 2[a] | 1.5 | 4.8 | 3.0 | 9.6 |
| Expt 3[a] | 2.5 | 5.0 | 4.1 | 12.6 |
| Expt 4[a] | 2.0 | 2.7 | 12.0 | 12.0 |
| Expt 5[a] | 2.5 | 7.1 | 10.7 | 13.2 |
| Expt 6[a] | 2.0 | 4.0 | 4.0 | 10.5 |
| HX47[b] | 8 | 13 | 17 | 21 |
| HX47[b] | 7 | 8 | 11 | 18 |
| HX34[b] | 6 | 10 | 14 | 18 |
| HX118[b] | 8 | 8 | 8 | 14 |

[a]B16 melanoma in C57B1 mice. [b]Three human melanoma xenografts in immune-deprived mice.

**Figure 2** Mean body weight (±s.e.m.) expressed as a ration of weight on day 0. ● = control, ▲ = BCNU 35 mg $kg^{-1}$, ○ = TNF $2.5 \times 10^5$ U $kg^{-1}$, ■ = combination TNF + BCNU.

**Figure 3** Effect of TNF and BCNU (±s.e.m.) on peripheral white cell count. ● = control, ▲ = TNF $2.5 \times 10^5$ U $kg^{-1}$, ○ = BCNU 35 mg $kg^{-1}$, ■ = combination TNF + BCNU.

Immune deprived mice bearing human melanoma xenografts tolerated higher doses of TNF than C57 BL mice with B16 melanomas (Table I) and a dose of $1 \times 10^6$ U (489 ng) per kg was used in xenograft experiments. The results of experiments on three different xenografts are shown in Table II. The delay in doubling time was not significant with TNF alone ($P = 0.057$) but BCNU did cause significant growth delay ($P = 0.029$). There was significant growth delay with combination treatment ($P = 0.014$) and this was significantly longer than the delays seen with BCNU alone ($P = 0.029$). When the schedule of TNF was altered to $1 \times 10^6$ U (489 ng) per kg b.d. there was further enhancement of growth delay (Figure 4). In this experiment the dose of BCNU was constant. Figure 4a represents TNF once daily and Figure 4b shows the enhancement of growth delay with twice daily TNF. No toxicity was observed in these animals.

### Discussion

These results have shown that the cytotoxic activity of BCNU on B16 melanoma cells *in vitro* was enhanced by the addition of TNF across a range of concentrations of BCNU. TNF in combination with BCNU increased the growth delay of the B16 melanoma compared with that seen using either BCNU or TNF alone.

The addition of TNF to BCNU did not result in increased toxicity as measured by the peripheral blood count. Although mice receiving combination treatment had weight loss of up to 20%, there were no toxic deaths, and all mice regained their starting weight by the end of the experiment. Enhanced growth delay in the absence of significant toxicity represents an improvement in the therapeutic index. Weight gain was concurrent with tumour regrowth and may suggest that factors involved in mediating tumour regression may also be involved mediating weight loss in these animals. TNF itself has been implicated as a mediator of cachexia (Cerami *et al.*, 1985) but no significant weight loss was seen in the group receiving TNF alone compared with untreated control animals.

The enhanced antitumour effect with combination treatment was unlikely to be caused by altered BCNU pharmacokinetics as the addition of TNF did not affect the pattern of myelosuppression after BCNU. TNF may affect tumour vasculature (Nawroth & Stern, 1986) and hence drug permeability, but in these experiments BCNU was given with the initial dose of TNF making this explanation less likely. The *in vitro* data suggest that TNF has a direct effect on the cytotoxicity of BCNU. Similar potentiation of drug-induced cytotoxicity by TNF *in vitro* and *in vivo* has been reported

**Figure 4** Tumour growth delay of (±s.e.m.) human melanoma xenograft with BCNU and TNF. **a**, TNF $1 \times 10^6$ U $kg^{-1}$ daily; ———. **b**, TNF $1 \times 10^6$ U $kg^{-1}$ b.d. – – –. ● = control, ○ = BCNU 35 mg $kg^{-1}$, ▲ = TNF, ■ = combination TNF + BCNU.

(Alexander *et al.*, 1987; Das *et al.*, 1989; Regenass *et al.*, 1987). It is important to assess such activity *in vivo* as well as *in vitro* to confirm activity within the host and to exclude significant toxicity. Such an interaction has been demonstrated here *in vivo* for BCNU and TNF in syngeneic tumours and in xenografts. Although the mechanism may be related to enhanced direct cytotoxicity, *in vivo* host factors such as tumour vasculature may also be relevant.

Tumour regression in C57 BL mice occurred at TNF doses close to the maximum tolerated dose (MTD) for tumour bearing animals. With the human melanoma xenografts the dose of TNF had to be increased nearer to the MTD for this strain, to achieve growth delay. It appears that increased exposure to TNF *in vivo* may be important as a twice daily dose schedule was more effective. This may have a pharmacokinetic basis as TNF is rapidly cleared from serum (Jones & Selby, 1990).

Data from phase I trials suggest that the therapeutic index of TNF in man is small (Selby *et al.*, 1987) and toxicity may preclude its use in cancer patients. Certainly our data suggest excess toxicity in tumour bearing animals and this may argue for the use of TNF and other biological therapies in the setting of minimal disease. The clinical application of TNF may be increased by its use in combination therapy with cytotoxic drugs. The mechanisms of such interactions require further evaluation. However, even with combination therapy it is likely that the necessary dose of TNF will be close to the maximum tolerated dose and combination therapy should be approached cautiously because of the possibility of increased toxicity to the host. A phase III randomised trial of BCNU versus BCNU and TNF in patients with advanced melanoma is now underway at the Royal Marsden Hospital to investigate the efficacy of TNF in combination with BCNU in patients with disseminated malignant melanoma.

This work was supported by the Cancer Research Campaign of Great Britain. We thank Miss Julie Bingley for typing the manuscript.

### References

AHMANN, D.L. (1976). Nitrosoureas in the management of disseminated malignant melanoma. *Cancer Treat. Rep.*, **60**, 747.

ALEXANDER, R.B., NELSON, W.G. & COFFEY, D.S. (1987). Synergistic enhancement by tumor necrosis factor of in vitro cytotoxicity from chemotherapeutic drugs targeted at DNA topoisomerase II. *Cancer Res.*, **47**, 2403.

BALKWILL, F.R., LEE, A., ALDAM, G. & 4 others (1986). Human tumor xenografts treated with recombinant human tumor necrosis factor alone or in combination with interferons. *Cancer Res.*, **46**, 3990.

BROUCKAERT, P.G.G., LEROUX ROELS, G.G., GUISEZ, Y., TAVERNIER, J. & FIERS, W. (1986). In vivo antitumour activity of recombinant human and murine TNF, alone and in combination with murine IFN-gamma, on a syngeneic murine melanoma. *Int. J. Cancer*, **38**, 763.

CARSWELL, E.A., OLD, L.J., KASSEL, R.A., GREEN, S., FIORE, N. & WILLIAMSON, B. (1975). An endotoxin-induced serum factor that causes necrosis of tumours. *Proc. Natl Acad. Sci. USA*, **72**, 3666.

CERAMI, A., IKEDA, Y., LE TRANG, N., HOTEZ, P.J. & BEUTLER, B. (1985). Weight loss associated with an endotoxin-induced mediator from peritoneal macrophages: the role of cachectin (tumor necrosis factor). *Immunol. Lett.*, **11**, 173.

COMIS, R.L. (1976). DTIC (NSC-45388) in malignant melanoma: a perspective. *Cancer Treat. Rep.*, **60**, 165.

CREAGEN, E.T., AHMANN, D.L., FRYTAL, S., LONG, H.J., CHANG, M.N. & ITRI, L.M. (1987). Three consecutive Phase II studies of recombinant interferon alfa-2a in advanced malignant melanoma. *Cancer*, **59**, 638.

CREASEY, A., REYNOLDS, M.R. & LAIRD, W. (1986). Cures and partial regressions of murine and human tumors by recombinant human tumor necrosis factor. *Cancer Res.*, **46**, 5687.

DAS, A.K., WALTHER, P.J., BUCKLEY, N.J. & POULTON, S.H.M. (1989). Recombinant human tumor necrosis factor alone and with chemotherapeutic agents. *Arch. Surg.*, **124**, 107.

FRANSEN, L., RUYSSHAERT, M.R., VAN DER HEYDEN, J. & FIERS, W. (1975). Recombinant tumour necrosis factor, species specificity for a variety of human and transformed cell lines. *Cell. Immunol.*, **100**, 260.

HARANAKA, K., SATOMI, N. & SAKAURAI, A. (1984). Antitumor activity of murine tumor necrosis factor (TNF) against transplanted murine tumors and heterotransplanted human tumors in nude mice. *Int. J. Cancer*, **34**, 263.

HELSON, L., GREEN, S., CARSWELL, E.A. & OLD, L.J. (1975). Effect of tumour necrosis factor on cultured human melanoma cells. *Nature*, **258**, 731.

JONES, A.L. & SELBY, P.J. (1990). Tumour necrosis factor: clinical relevance. *Cancer Surv.* (in the press).

KOPPER, L. & STEEL, G.G. (1975). The therapeutic response of three human tumour lines maintained in immune-suppressed mice. *Cancer Res.*, **35**, 2704.

LAKHANI, S., SELBY, P., BLISS, J.M., PERREN, T.J., GORE, M.G. & MCELWAIN, T.J. (1990). Chemotherapy for malignant melanoma; combinations and high doses produce more responses without survival benefit. *Br. J. Cancer* (in the press).

MANNEL, D.N., MOORE, R.N. & MERGENHAGEN, S.E. (1980). Macrophages as a source of tumouricidal activity (tumour necrotising factor). *Infect. Immunol.*, **30**, 523.

MARMENOUT, A., FRANSEN, L., TAVERNIERE, J. & 10 others (1985). Molecular cloning and expression of human tumour necrosis factor and comparison with mouse tumour necrosis factor. *Eur. J. Biochem.*, **152**, 515.

MARSH, J.C. (1976). The effects of cancer chemotherapeutic agents on normal hematopoietic cells: a review. *Cancer Res.*, **36**, 1853.

NAWROTH, P.P. & STERN, D.M. (1986). Modulation of endothelial cell hemostatic properties by tumour necrosis factor. *J. Exp. Med.*, **123**, 16.

PENNICA, D., NEDWIN, G.E., HAYFLICK, J.S. & 6 others (1984). Human tumour necrosis factor: precursor structure, expression and homology to lymphotoxin. *Nature*, **312**, 724.

REGENASS, V., MULLER, M., CURSCHELLAS, E. & MALTER, A. (1987). Antitumour effects of tumour necrosis factor in combination with chemotherapeutic agents. *Int. J. Cancer*, **39**, 266.

ROSENBERG, S.A., RAPP, H., TERRY, W. & 7 others (1982). Intralesional BCG therapy of patients with primary stage 1 melanoma. In *Immunotherapy of Human Cancer*, Terry, W.D. & Rosenberg, S.A. (eds) p. 289. Elsevier/North Holland: New York.

ROSENBERG, S.A., LOTZE, M.T., MUUL, L.M. & 10 others (1987). A progress report on the treatment of 157 patients with advanced cancer using lymphokine activated killer cells and interleukin-2 or high dose interleukin-2 alone. *N. Engl. J. Med.*, **316**, 889.

SELBY, P., HOBBS, S., FEARON, K. & 8 others (1987). Tumour necrosis factor in man: clinical and biological observations. *Br. J. Cancer*, **56**, 803.

ZACHARCHUK, C.M., DRYSDALE, B.E., MAYER, M.M. & SHIN, H.S. (1983). Macrophage-mediated cytotoxicity: role of a soluble macrophage cytotoxic factor similar to lymphotoxin and tumor necrosis factor. *Proc. Natl Acad. Sci. USA*, **80**, 6341.